# Living without pain: A 10-year study of congenital insensitivity to pain with anhidrosis

**DOI:** 10.1038/s41390-024-03565-x

**Published:** 2024-10-25

**Authors:** Shai Shlomi klaitman, Galina ling, Eyal Kristal, Odeya David, Siham Elamour, Eli Hershkovitz, Eduard Ling

**Affiliations:** 1 Faculty of Health Sciences, Ben Gurion University Medical Center, Beer Sheva, Israel; 2 Soroka University Medical Center, Rager Avenue, Beer Sheva, Israel; 3 Pediatric Ambulatory Unit, Soroka University Medical Center, Rager Avenue, Beer Sheva, Israel; 4 Immunology Unit, Soroka University Medical Center, Beer Sheva, Israel; 5 Pediatric Endocrinology And Diabetes Unit, Assuta Ashdod Hospital, Ashdod, Israel; 6 The Pediatric Infectious Disease Unit, Soroka University Medical Center, Beer-Sheva, Israel; 7 Department of Pediatrics D, Saban Center of Pediatrics, Soroka University Medical Center, Beer Sheva, Israel; 8 Pediatric Endocrinology Unit, Saban Center of Pediatrics, Soroka University Medical Center, Beer Sheva, Israel; 9 Rheumatology Clinic, Clalit Health Services, Southern District, Beer Sheva, Israel

## Abstract

**Background:**

Congenital Insensitivity to Pain with Anhidrosis (CIPA) is a rare hereditary neuropathy caused by NTRK1 gene mutations, predisposing patients to recurrent infections and chronic wounds. Long-term studies on microbial and clinical outcomes in CIPA are limited. This study presents analysis of infection patterns, antibiotic resistance, and clinical outcomes in CIPA patients.

**Methods:**

A comprehensive ten-year retrospective cohort study was conducted at Soroka University Medical Center, Beer Sheva, Israel, from January 2014 to January 2023. Electronic medical records were reviewed to identify 63 CIPA patients, all were of consanguineous Bedouin families. Data collection included demographic details, clinical presentations, genetic analysis, documentation of infections, wound sites, hospital duration, and surgical interventions.

**Results:**

*Staphylococcus aureus*, notably methicillin-resistant, dominated, with Gram-negative bacteria common in lower limbs. The study noted reduced extended-spectrum beta-lactamase bacteria and linked demographic factors to infection traits, antibiotic resistance, and surgical needs.

**Conclusion:**

This study provides valuable insights into the clinical and microbial patterns of CIPA, highlighting dynamic shifts in microbial compositions and antibiotic resistance profiles over time. *Staphylococcus aureus*, and Gram-negative bacteria particularly in lower limb infections, pose significant challenges in patient management. The findings underscore the importance of tailored approaches to address evolving microbial profiles and optimize patient care in CIPA.

**Impact:**

- Key Message: This is the largest cohort study on CIPA to date, highlighting the dominance of Staphylococcus aureus, including methicillin-resistant strains, and significant Gram-negative bacterial infections in lower limbs.
- Contribution to Literature: It draws parallels between infection dynamics in CIPA and diabetic foot ulcers, emphasizing similar challenges due to neuropathy and ischemia, enhancing understanding of infection susceptibility and management in neuropathic conditions.
- Impact: The findings inform clinical practices by detailing infection and resistance patterns, supporting the development of targeted treatment strategies to improve outcomes for CIPA and similar conditions.

## Introduction

Congenital insensitivity to pain with anhidrosis (CIPA), also known as hereditary sensory and autonomic neuropathy type 4 (HSAN-4), is a rare autosomal recessive disorder (MIM number 256800^1^) characterized by pain insensitivity, recurrent episodes of infections and unexplained fever, anhidrosis (inability to sweat), and varying degrees of intellectual disability. This condition arises from mutations in the NTRK1(TRKA) gene on chromosome 1q21-q22, encoding the tropomyosin receptor kinase A (trkA), an Nerve growth factor (NGF) receptor.^2^ TrkA’s interaction with NGF activates the RAS/MAPK pathway, which is crucial for cellular proliferation and growth through Erk signaling.^3^ TrkA also plays a vital role in the musculoskeletal system, influencing the function of fibroblasts, keratinocytes, endothelial cells, and chondrocyte progenitors that are essential for wound healing, bone development, and repair.^4–6^

Patients with CIPA face increased risks of injuries and complications like osteomyelitis, septic arthritis, other arthropathies, fractures, often requiring recurrent medical interventions.^7–9^ The disorder is particularly prevalent in Japanese,^10^ Israeli Bedouin,^2^ and Chinese populations,^11^ with notable frequencies due to genetic factors like consanguinity in certain communities. Research on CIPA is limited, with the majority of literature comprising case reports that primarily document new cases and patient phenotypes.^12^ As such, the global prevalence of the disorder remains poorly understood, with the global incidence being estimated at about 1 per 125 million newborn.^13^

We conducted a comprehensive ten-year retrospective cohort of Congenital Insensitivity to Pain with Anhidrosis (CIPA) patients, focusing on the evolution of clinical presentations. In our study, we aimed to analyze longitudinal clinical and microbial data in CIPA patients, assess the emergence of pathogen and antibiotic resistance profiles, and examine the potential influence of escalating antibiotic resistance rate on the disease course and patient outcome over time.

## Methods

### Study design and setting

This retrospective cohort study was conducted at Soroka University Medical Center, Beer Sheva, Israel, spanning a decade from January 2014 to January 2023. The study was designed to observe clinical and microbial patterns in patients diagnosed with Congenital Insensitivity to Pain with Anhidrosis (CIPA).

### Ethical approval

The research protocol was approved by the Institutional Review Board (IRB) at Soroka University Medical Center, in accordance with ethical standards and patient privacy regulations as per the Helsinki Declaration.

### Participants

Patients diagnosed with CIPA were retrospectively identified from electronic medical records using the International Classification of Diseases, Ninth Revision (ICD-9) code for congenital insensitivity to pain and anhidrosis. For study inclusion, (i) a disease-causing mutation for CIPA was confirmed through genetic testing, or (ii) the patients exhibited key clinical hallmarks of CIPA (lack of pain sensation, inability to sweat, recurrent fevers, self-mutilation injuries) and had a sibling with a previous CIPA diagnosis or were from a tribe known to have a high CIPA prevalence. Further inclusion criteria encompassed hallmark features of CIPA such as lack of response to noxious stimuli, anhidrosis, recurrent fevers, and self-mutilation. Additional clinical signs included pronounced xeroderma with hyperkeratosis and fissuring, especially on the palmar and plantar regions, delayed wound healing, deep-seated infections, and osteomyelitis. Exclusion criteria were the following: patients with a distinct genotype and phenotype, patients not agreeing to participate in the study, inability to provide informed consent, and missing or incomplete data on outcome measures.

### Treatment protocols

For patients who had not previously experienced infections, we began with empirical antibiotic therapy, primarily targeting *Staphylococcus aureus*. As the disease progressed and specific bacterial infections emerged, antibiotic selection was meticulously guided by the patient’s bacteriological profiles and resistance patterns identified through prior cultures. In each episode of infection, the initial empirical antibiotic was adjusted based on the sensitivity results as soon as they became available. A crucial consideration in selecting antibiotics was the patient’s medical history, particularly any known allergies to certain antibiotics. This personalized approach ensured that each patient received the most effective treatment tailored to their needs, while also minimizing the risk of adverse reactions.

### Data collection

Patient charts were reviewed using Chameleon Electronic Medical Records (EMR) Software.^14^ Data collected included demographic details, clinical presentations, genetic analysis with next-generation sequencing (NGS), documentation of infections, wound sites, hospital duration and details of any surgical interventions.

### Statistical analysis

R software version 4.3.0 with the ‘expss’ package for data presentation^15^ was used. An assumption of normal distribution in the absence of equal variances was made for the purpose of this study. Power analysis was carried out on GPower 3.1.9.7 and statistical significance was set at an alpha level of 0.05. The Bonferroni^16^ correction was applied to account for multiple comparisons. Data were analyzed using z-tests, and regression models to assess patient demographics, infection characteristics, and antibiotic resistance.

## Results

### Patients

Our study reviewed a group of 63 patients diagnosed with Congenital Insensitivity to Pain with Anhidrosis (CIPA), all were of consanguineous Bedouin families. This cohort was composed of 33 male and 30 female participants, with an age range extending from 6 months to 33.4 years. 4 patients were aged 0–2 years, 19 patients were aged 2–10 years, 16 patients were aged 10–18 years, and 24 patients were aged >18 years. Throughout the duration of the study, from January 2014 to January 2023, there were 19 new diagnoses of CIPA, and we documented 9 fatalities. Of these fatalities, 2 had experienced septic shock and 2 had experience cardiac arrest post-surgery likely attributable to dysautonomia. Genetic testing was performed on 23 patients who were found to be homozygous for the NTRK1 1926insT mutation. Genetic analysis was not conducted on the remaining 40 participants for reasons such as established prevalence in specific tribes, diagnosed siblings, or lack of consent. One patient was excluded from the study. This patient harbored the NTRK1 c.1860-1861insT mutation^17^ that is prevalent among Palestinian patients, and exhibited a phenotype characterized by recurrent seizures.

### Pathogen distribution and bacterial growth patterns in clinical infections

Table 1 outlines the occurrence of bacterial pathogens within 1459 documented infection events, sorted according to the anatomical site of infection. Gram-negative bacteria were responsible for 40.1% of these cases, of which 15.2% exhibited resistance characteristics associated with Extended-spectrum beta-lactamases (ESBL). The primary Gram-negative pathogens (data not shown), were *Pseudomonas* spp., contributing to 13% of the infections, *Morganella* spp. at 5.7%, *Enterobacter* spp. at 3.5%, *Proteus* spp. at 3.5%, *Klebsiella* spp. at 2.2%. with additional unspecified Gram-negative bacteria comprising the rest of the infections. 0.6% of events involved co-infection with *Candida* spp.

**Table 1:** Distribution of infection episodes by clinical site.

	#Total (%) (*n* = 1459)	Blood	Lower Limb		Upper Limb		Other	
			Deep	Superficial	Deep	Superficial	Deep	Superficial
		A	B	C	D	E	F	G
Gram-negative	34.0	12.8 ^–^	33.7	38.3*^,E^	20.9	13.0 ^–^	27.8	28.9^E^
Gram-negative ESBL +	6.1	5.1	2.0 ^–^	7.0*^,B^	0	3.3	22.2	11.1^E^
Gram-positive	26.0	38.5	30.6	24.9	27.9	23.5	16.7	26.7
Other	0.7	0	0	1.0	0	0	0	0
staphylococcus aureus	17.1	23.1	14.8	14.5	32.6*^,F^	35.8*^,C,G^	5.6	17.8
staphylococcus aureus MRSA +	16.1	20.5	18.9	14.3	18.6	24.4*^,C^	27.7	15.5
#Total cases	100%	2.5%	13.5%	67.5%	3%	8.5%	2%	3%

*(*p* < 0.05), Statistically significant differences between groups (anatomical sites or bacterial culture types) are displayed using letters assigned to each group. For example, letter ‘A’ is noted for the values significantly different from A column.

"–" sign indicates that a group has significantly less prevalence at *p* < 0.05 compared to other groups at the same row.

*Staphylococcus aureus* was identified as the most common pathogen across all categories, accounting for 33.2% of the total cases, with methicillin-resistant *Staphylococcus aureus* (MRSA) making up 48.5% of the *Staphylococcus aureus* infections. Gram-positive bacteria, other than *Staphylococcus spp*. were responsible for 26% of the total cases, with *Streptococcus* spp. being the second most prevalent at 17.5% of all bacterial infections documented.

Of utmost importance to note, significant (*p < 0.05) differences were observed upon analysis of infections in relation to the anatomical site. Lower limb infections accounted for the majority of cases with 81% (*n* = 1179) occurring in superficial and deep sites, with gram-negative bacteria as the most dominant isolate. The prevalence of gram-negative bacterial infection at superficial wounds of lower limb was significantly higher when compared to superficial wounds in upper limb (*p < 0.05). In contrast, *Staphylococcus aureus* was more frequently isolated from upper limb infections, representing 35.8% of isolates in superficial wounds of the upper limbs, which was significantly higher than superficial wounds of lower limb wounds or superficial wounds in other anatomical sites (*p* < 0.05). Gram-negative bacteria were cultured significantly less from blood and superficial infections of upper limb (*p < 0.05).

Analysis of the bacterial growth patterns revealed that 61.3% of the cultures displayed polymicrobial growth, whereas 38.7% demonstrated isolated bacterial growth. *Staphylococcus aureus* was more likely to be cultured as a sole organism compared to other bacteria (*p < 0.05).

The study included an assessment of the microbial compositions in wound infections, with an emphasis on the relationship between the depth and anatomical location of the wound (either on the lower or upper extremities) and the nature of bacterial colonization (either isolated or polymicrobial) as detailed in Table 2. While analyzing the deep wounds, regardless of their location on the lower or upper limbs, a stiking observation of monomicrobial bacterial colonization was made. In contrast, superficial wounds located on the lower extremities tended to present with polymicrobial colonization (*p < 0.05). On the other hand, superficial wounds of the upper extremities were characterized by monomicrobial colonization (*p < 0.05). An additional noteworthy finding was the detection of isolated bacterial growth in blood cultures, with statistical significance (*p < 0.05).

**Table 2:** Incidence of monomicrobial vs. polymicrobial growth in different site locations.

	#Total	Monomicrobial	Polymicrobial
		A	B
Lower limb: Deep	13.5	18.3*^,B^	10.4 ^–^
Lower limb: Superficial	67.5	52.7	76.6*^,A^
Upper limb: Deep	3	5.3*^,B^	1.5 ^–^
Upper limb: Superficial	8.5	13.7*^,B^	5.1 ^–^
Blood	2.5	5.5*^,B^	0.9 ^–^
Other	5	4.5	5.5
#Total cases	100%	39%	61%

*(*p* < 0.05), Statistically significant differences between groups (anatomical sites or bacterial culture types) are displayed using letters assigned to each group. For example, letter ‘A’ is noted for the values significantly different from 'A' column.

"–" sign indicates that a group has significantly less prevalence at *p* < 0.05 compared to other groups at the same row.

### Dynamics of bacterial infection patterns and surgical interventions

Throughout the duration of the study, there was a significant decline in the incidence of extended-spectrum beta-lactamase (ESBL)-producing bacteria and in ESBL carriage (*p < 0.05), while the incidence of MRSA carriage remained consistent. Additionally, there was an observed increase in superficial wounds of the lower extremities, in contrast to a decrease in superficial wounds of the upper extremities (*p < 0.05).

Throughout our study period, the most commonly performed surgical procedures were incision and drainage, constituting 18.5% of all surgeries, with amputation and arthrotomy following at 6% and 4% respectively. Notably, there was a significant decrease in the total number of surgeries conducted as the study progressed (*p < 0.05).

### Regression analysis of patient demographics, infection characteristics, and clinical outcomes in antibiotic resistance

We used the age group 0–2 years as the reference category in our regression analyses to serve as a baseline for comparison. This choice was informed by several factors. Firstly, individuals within this age range are typically not mobile, reducing the likelihood of injuries that could influence the outcomes under investigation. Furthermore, infants and toddlers in this category are less prone to existing infections in their wounds and are unlikely to require surgical interventions, thus minimizing potential confounding variables related to wound healing or postoperative care.

Male sex was associated with lower odds of gram-negative bacterial wound infections (OR 0.709; 95% CI, 0.563–0.891; **p < 0.01), but higher odds of *Staphylococcus aureus* infections (OR 1.337; 95% CI, 1.001–1.791; *p* < 0.05) (Table 3). Patients aged ≥2 years had over 11 times the odds of developing gram-negative infections compared to the 0–2 year group (*p < 0.05). In contrast, patients aged ≥2 years had significantly lower odds of *Staphylococcus aureus* infections than the youngest cohort (OR 0.2–0.24; **p < 0.01 and ***p < 0.001).

**Table 3:** Age and sex predictors of harboring antibiotic-resistant infections.

Predictor	Gram Negative OR (95% CI)	Gram Negative ESBL OR (95% CI)	Gram Positive OR (95% CI)	staphylococcus aureus OR (95% CI)	MRSA OR (95% CI)
Sex: Male	0.709** [0.563, 0.891]	0.745 [0.470, 1.170]	1.254 [0.980, 1.606]	1.337* [1.001, 1.791]	1.040 [0.775, 1.396]
Age: 2–10 years	11.008* [2.273, 198.178]	0.749 [0.210, 4.773]	1.439 [0.525, 5.049]	0.203*** [0.084, 0.498]	0.776 [0.282, 2.732]
Age: 10–18 years	11.223* [2.297, 202.616]	0.459 [0.120, 3.020]	1.597 [0.576, 5.641]	0.210*** [0.085, 0.523]	0.797 [0.284, 2.837]
Age: > 18 years	11.121* [2.203, 202.873]	0.538 [0.119, 3.804]	1.142 [0.387, 4.194]	0.243** [0.092, 0.641]	1.052 [0.354, 3.880]

^*^p < 0.05, **p < 0.01, ***p < 0.001.

Male sex was associated with a lower likelihood of polymicrobial infections (OR 0.758; 95% CI, 0.596–0.963; *p < 0.05). The 2–10 years age group had a higher likelihood of polymicrobial infections (OR 3.818; 95% CI, 1.311–13.932; *p < 0.05). The presence of *Staphylococcus aureus* bacteria MSSA+ or MRSA+ was associated with a lower likelihood of polymicrobial infections (OR 0.340; 95% CI, 0.243–0.475; ***p < 0.001) and (OR 0.353; 95% CI, 0.251–0.493; ***p < 0.001), respectively. Compared to the upper torso location, infections in the lower limbs (OR 6.354; 95% CI, 2.929–15.393; ***p < 0.001) had a higher likelihood of polymicrobial infections.

All age groups – 2–10 years (OR 34.179; 95% CI, 10.44–108.91; ***p < 0.001), 10–18 years (OR 49.405; 95% CI, 11.621–259.62; ***p < 0.001), and >18 years (OR 10.957; 95% CI, 2.765–48.79; ***p < 0.001) had significantly higher odds of requiring surgery compared to the reference age group ( < 2 years). Infection site also emerges as a predictor in this model. Deep infections had significantly higher odds of requiring surgery (OR 12.196; 95% CI, 1.235-271.445; *p < 0.05), while superficial infections and other sites- including but not limited to urine, ear, throat, eyes, did not demonstrate statistically significant associations.

A multivariable linear regression model applied to predict the hospital duration by sex, pathogens, and polymicrobial status (Table 4) showed that the presence of ESBL+ Gram-negatives was associated with longer hospital duration ( + 5.989 days, ***p < 0.001). In contrast, *Staphylococcus aureus* infections and polymicrobial infections were associated with shorter hospital stay duration (−2.054 days, *p < 0.05, and −5.084 days, ***p < 0.001, respectively).

**Table 4:** Linear regression model predicting hospital duration by sex, pathogens, and polymicrobial status.

	Hospital duration (Days)
Sex: Male	1.185 [ − 0.153, 2.523]
Bacteria type: Gram-negative ESBL+	5.989^***^ [3.081, 8.898]
Bacteria type: Gram-positive	−0.919 [ − 2.647, 0.808]
Bacteria type: Other	3.714 [ − 4.355, 11.783]
Bacteria type: staphylococcus aureus	−2.054^*^ [−4.064, −0.044]
Bacteria type: MRSA+	1.96 [ − 0.072, 4.003]
Bacteria culture: Polymicrobial	−5.084^***^[ − 6.500, −3.668]

^*^p < 0.05, ***p < 0.001.

## Discussion

This 10-year retrospective cohort study of 63 patients with Congenital Insensitivity to Pain with Anhidrosis (CIPA) provides valuable insights into the clinical and microbial patterns in this rare disorder. Our analysis of nearly 1500 documented infections revealed shifting microbial compositions and antibiotic resistance profiles over time, with implications for patient outcomes.

Similar to earlier reports that highlighted gram-positive bacteria as the main pathological agents,^8,18^ our study confirmed that *Staphylococcus aureus* was the most common bacterium isolated, accounting for 33.2% of cases (Table 1). While MRSA represented 48.5% of *Staphylococcus aureus* isolates. *Streptococcus* spp. were the second most frequent Gram-positive pathogen, comprising 17.5% of all documented bacterial infections. Gram-negative pathogens were isolated in 40.1% of all cultures, whereas they constituted more than 80% of the pathogens isolated from the lower limbs of CIPA patients in this study. Of importance to note that *Pseudomonas* spp. was identified in 13% of all cultures, thus being the third most frequent pathogen, following *Staphylococcus aureus* and *Streptococcus* spp.

We hypothesize that the neuropathic and ischemic changes inherent to the lower limbs of CIPA patients may provide a milieu beneficial to infections, similar to that observed in diabetic foot ulcers (DFUs), where a combination of repeated trauma, neuropathy, and ischemia analogously elevates the risk of infections. In a meta-analysis conducted by Macdonald et al.^19^ on the microbiology of diabetic foot infections, there is an apparent dichotomy in the bacterial spectrum of DFUs between high and low-income countries. Predominantly, Gram-negative bacteria are more frequently identified in lower-income nations, whereas Gram-positive bacteria are more common in higher-income settings. Consistently, our study revealed a predominance of Gram-negative bacteria in lower limbs of CIPA patient cohort from Israel, a high-income country. This deviation could be explained by the unique socioeconomic conditions prevalent among the Bedouin communities in southern Israel, where poverty is higher than in any other segment of the population.^20^ A significant proportion of these communities live in poverty, which is reflected in their environmental conditions, footwear choices, and hygiene practices.

A key finding of our study is the high prevalence of *Staphylococcus aureus* and MRSA in infection sites. Notably, a similar observation was made by Fruchtman et al.^8^ The authors collected data from children with CIPA in Southern Israel and from years 1991–2005. They reported that the most frequent pathogen was *Staphylococcus aureus* at infection sites of the skin and skeleton, with a fair subset being treatment-resistant. We propose that the observed phenomenon can be ascribed to defects in host immunity specific to CIPA, which may encompass a range of effects related to the NGFβ-TRKA pathway. Notably, these effects could significantly impact innate immunity against Staphylococcus aureus infection. The intricate interplay between NGF-TRKA signaling and innate immunity, particularly in the context of Staphylococcus aureus infections, alongside the compromised chemotactic function of neutrophils in CIPA patients, underscores their heightened vulnerability to infections and the prolonged healing of wounds.^21,22^

In spite of comprehensive analysis of microbiological and clinical data of a large cohort of CIPA patients, our study is not devoid of limitations. CIPA is a very rare disorder, rendering the sampling of eligible cases challenging. For this purpose, we extracted data from the Chameleon Electronic Medical Records software. This software documents the medical records of the patient admitted to the hospitals or of the patients visiting the Hospital Ambulatory Services Unit.^14^ The wound cultures are obtained only during the hospital facilities visit, but not in the outpatient clinics. Hospitalized cases may not be representative of non-hospitalized CIPA cases. As mentioned earlier, poverty in Bedouin communities is higher than other segments of the Israeli population. It is, thus, possible that cases with significantly poor access to healthcare were inadvertently not included in the present study, which would only impact on the overall generalizability of our findings.

Patients that did not consent to genetic testing were included if they exhibited clinical features of CIPA, had siblings with a CIPA diagnosis, or came from a tribe with a known CIPA prevalence. This omission could have acted as a source of bias, as CIPA patients can harbor different NTRK1 mutations and variants,^23^ which may or may not have been associated with different clinical and microbial patterns. This is information that we could not ethically extract from patients that did not consent to genetic testing. Finally, there were variables that we could not incorporate into our analysis of clinical and microbial parameters of infection sites. Of note, our study did not account for persistence of infection in specific anatomical sites of CIPA patients, duration of antibiotic treatments, or specific antibiotic regimes. Among other potential limitations of electronic health records, missing data and incorrect data entered by clinicians should be listed.

In addition to the study’s methodological limitations, it is crucial to highlight the proactive patient management approach implemented throughout the study period. All patients were enrolled in a specialized multidisciplinary day clinic designed to provide comprehensive care and close monitoring. The clinic’s team consisted of a pediatrician, an infectious disease specialist, an orthopedic surgeon, an immunologist, a gastroenterologist, and a dietitian. Regular follow-ups were scheduled to assess the patients’ clinical status, manage ongoing treatments, and adjust care plans as needed to detect wound infections as early as possible. Notably, these consistent and coordinated follow-ups significantly reduced the number of hospitalizations and prevented unnecessary surgical interventions, demonstrating the effectiveness of proactive and integrated care management.

Given the bacteriological profiles that often included multiple antibiotic-resistant organisms, the clinic implemented strict protocols to prevent cross-contamination among patients. Specifically, those who had not yet acquired resistant bacteria were seen separately to minimize the risk of infection within the patient cohort. This aspect of patient care, although not the primary focus of the study, underscores the importance of comprehensive and multidisciplinary approaches in managing complex conditions like CIPA while maintaining stringent infection control measures.

## Conclusion

In this extensive 10-year retrospective cohort study, we meticulously examined the clinical and microbial landscapes of Congenital Insensitivity to Pain with Anhidrosis (CIPA), shedding light on its nuanced complexities and offering crucial insights into therapeutic strategies for managing this rare disorder. Our findings illuminate dynamic shifts in microbial compositions over time, with *Staphylococcus aureus* emerging as the predominant pathogen, accompanied by a concerning proportion of methicillin-resistant strains. Notably, Gram-negative bacteria, particularly prevalent in lower limb infections, present formidable challenges in patient care, underscoring the need for tailored therapeutic approaches.

Moreover, our regression analyses reveal demographic factors associated with infection characteristics and clinical outcomes, offering valuable prognostic insights and guiding principles for personalized treatment strategies. Understanding these predictors of antibiotic resistance and surgical intervention needs is paramount in optimizing patient care and improving long-term outcomes.

## Data Availability

The data that support the findings of this study are available on request from the corresponding author, Dr. Shai Shlomi Klaitman (SK). The data are not publicly available due to concerns about compromising the privacy of research participants.
